# Loneliness and depressive symptoms differ by sexual orientation and gender identity during physical distancing measures in response to COVID‐19 pandemic in Germany

**DOI:** 10.1111/aphw.12376

**Published:** 2022-06-06

**Authors:** Wolfram J. Herrmann, Philip Oeser, Pichit Buspavanich, Sonia Lech, Maximilian Berger, Paul Gellert

**Affiliations:** ^1^ Institute of General Practice Charité ‐ Universitätsmedizin Berlin Berlin Germany; ^2^ Münster School of Health FH Münster Münster Germany; ^3^ Gender in Medicine (GiM) Charité ‐ Universitätsmedizin Berlin Berlin Germany; ^4^ Department of Psychiatry, Psychotherapy and Psychosomatics Brandenburg Medical School Theodor Fontane Neuruppin Germany; ^5^ Department of Psychiatry and Psychotherapy Charité ‐ Universitätsmedizin Berlin Berlin Germany; ^6^ Institute of Sexology and Sexual Medicine Charité – Universitätsmedizin Berlin Berlin Germany; ^7^ Faculty of Health Sciences Brandenburg Joint Faculty of the University of Potsdam, Brandenburg University of Technology Cottbus‐Senftenberg and Brandenburg Medical School Potsdam Germany; ^8^ Institute of Medical Sociology and Rehabilitation Science Charité ‐ Universitätsmedizin Berlin Berlin Germany

**Keywords:** depressive symptoms, LGBT, loneliness, minority stress, physical distancing

## Abstract

During the COVID‐19 pandemic, physical distancing measures to prevent transmission of the virus have been implemented. The effect of physical distancing measures on loneliness especially for vulnerable groups remained unclear. Thus, we aimed to investigate loneliness in relation with depressive symptoms among lesbian, gay, bisexual, trans, inter, asexual, and queer (LGBT) persons compared with cis‐heterosexual persons during the pandemic. We conducted an online survey during the first two waves of the COVID‐19 pandemic in Germany. The survey contained self‐categorizations regarding sexual orientation and gender identity, questions on loneliness, social contacts, depressive symptoms, and healthcare. Descriptive and regression analysis and propensity score matching across cohorts was conducted using R; 2641 participants took part in first wave of the survey and 4143 participants in the second wave. The proportion of lonely people was higher in the second wave compared with the first wave. LGBT persons were more lonely than cis‐heterosexual persons. In both waves, being LGBT was associated with depressive symptoms, but loneliness mediated the effect, even when adjusting for social contacts. Psychologists and other practitioners should be aware that LGBT clients might have an increased risk for loneliness and depressive symptoms and of the potential burden of the pandemic measures.

## BACKGROUND

### Physical distancing during the pandemic

The COVID‐19 pandemic led to physical distancing measures in many countries worldwide—often also called social distancing measures. The length and intensity of these physical distancing measures varied broadly between different states (Thu et al., 2020). However, core of all these measures was a reduction of direct physical contacts with other people to restrict the virus transmission. In Germany, physical distancing measures were implemented the first time in the end of March 2020 and varied over time and regionally with more intensified measures being in place in November 2020 and January/February 2021.

Physical distancing measures led to a significant reduction of infection rates and disease spreading (Bielecki et al., 2021; Daghriri & Ozmen, 2021). Conversely, the measures were associated with decreased levels in mental health and well‐being, which, in turn, were related with additional mental health problems such as stress, anxiety, or depressive symptoms (Torales et al., 2020; Tull et al., 2020) as well as with increased loneliness, especially among older adults (Heidinger & Richter, 2020; Macdonald & Hulur, 2021; van Tilburg et al., 2021). For young adults, physical activity implied a protective function against loneliness, whereas the need for meaningful, non‐virtual connections to peers during the pandemic was prevalent (Lippke et al., 2021).

### Loneliness and depressive symptoms

Loneliness has been defined as a state where a person perceives a discrepancy between their preferred and their actual social interactions and contacts (Cacioppo et al., 2015). This negative appraisal then is related with feelings of being alone. Loneliness further has been conceptualized as containing two facets, which are the feeling of missing an intimate relationship (i.e. emotional loneliness) and the feelings of missing a wider social network (i.e. social loneliness) (De Jong Gierveld & Tilburg, 2006). From the feeling and appraisal of loneliness, a lack of social integration has been distinguished (Cacioppo et al., 2015). Social integration refers to the “objective” number of contacts or the marital or living status and has been shown to be relatively distinct from the “subjective” concept of loneliness. Jenny de Jong Gierveld, in her seminal review on loneliness (De Jong Gierveld, 1998), pointed out that there are situations in which a person feels lonely despite having a lot of social contacts, and vice versa a person without social contacts may not feel lonely, e.g. if solitude was a conscious choice. However, empirical evidence suggests less social contacts and decreased social network use are linked with increased levels of loneliness (Lin et al., 2020).

In the general population, loneliness and depression are associated, which has been demonstrated in a recent systematic review (Erzen & Cikrikci, 2018). Further, there are studies that conceptualize loneliness as a mediator between exposure variables—in this case living urban/rural—and depressive symptoms (Giano et al., 2019).

### Lesbian, gay, bisexual, trans, inter, asexual, and queer (LGBT) as an especially vulnerable group

LGBT persons have previously been described to be more vulnerable for mental health problems compared to non‐LGBT population, experiencing higher levels of psychological distress, social anxiety, depression, and loneliness (Almeida et al., 2009; Anderssen et al., 2020; Eres et al., 2021; Hsieh & Liu, 2021; Hughes, 2018; Valentine & Shipherd, 2018; Wittgens et al., 2022). Also during the pandemic, lower levels of well‐being in LGBT compared with cis‐heterosexuals have been reported, as well as a higher risk for depressive symptoms in asexual, bisexual, non‐binary, and trans individuals (Buspavanich et al., 2021).

The main theory to explain these findings is the minority stress model. The minority stress model proposes (mental) health disparities as a consequence of social disadvantages, discrimination, and stigmatization related to race, gender, or sexual orientation. The model was constructed on gay men and differentiates between experienced actual discrimination, internalized homonegativity, and perceived stigma (Meyer, 2003). The minority stress model has been applied successfully to other sexual orientations and minorities (Cyrus, 2017; Hendricks & Testa, 2012). In several studies, minority stress explained higher levels of depression and suicidal ideation (Baams et al., 2015), loneliness (Kuyper & Fokkema, 2010), substance use (Goldbach et al., 2014), and social anxiety (Pachankis & Goldfried, 2006) in LGBT individuals.

### The relation of loneliness and depression in the LGBT population

Despite considerable research on the general population as already described, evidence on the relationship between loneliness and physical and mental health among the LGBT population remains rare: In addition to the findings of Giano et al. (2019) on the general population, Mereish et al. reported a mediating role of loneliness in the association between distal and proximal minority stressors and mental and physical distress among individuals of the LGBT community (Mereish & Poteat, 2015).

In the general population, elevated levels of loneliness have been reported during the COVID‐19 pandemic (Hwang et al., 2020) as well as an increase over the course of the pandemic (Killgore et al., 2020); in the LGBT population, loneliness has been especially reported for adolescents and younger adults (Fish et al., 2020; Gonzales et al., 2020; Salerno et al., 2020). However, research is sparse that looks into connecting the aspects of loneliness and depressive symptoms in LGBT persons. Taken together, a mediating role of loneliness between LGBT and depressive symptoms can be presumed according to the presented theory and evidence but has not yet been thoroughly tested in LGBT persons.

### Aim of the study

The aim of our study was to investigate loneliness and its relation to depressive symptoms for people with different sexual orientation and gender identity during the first two waves of the COVID‐19 pandemic. We measured the levels of loneliness and depressive symptoms in the Germany population in two separate cohorts in the first waves of the COVID‐19 pandemic; we hypothesised both loneliness and depressive symptoms to be higher late on in physical distancing measures (second wave) than in the beginning (first wave) because of the increasing length of physical distancing measures (Hypothesis 1). Further, in line with the minority stress model, we assumed LGBT persons to show elevated levels of loneliness and depressive symptoms compared to cis‐heterosexual persons (Hypothesis 2). Finally, we hypothesised loneliness to be related with depressive symptoms even when accounting for social contacts (i.e. face‐to‐face and telephone contacts) and age as potentially protective factors (Courtin & Knapp, 2017; Noguchi et al., 2021; Noone et al., 2020; Somes, 2021) (Hypothesis 3) and that loneliness plays a mediating role in the association of LGBT persons with depressive symptoms (Hypothesis 4).

## METHODS

We conducted an online survey with two waves of data acquisition. Data acquisition was open for 2 weeks in March/April 2020 (first wave) and January/February 2021 (second wave). Sampling was conducted as a snowball sampling, oversampling LGBT persons. We sent out the survey link via social media, as well as LGBT groups and organisations. In addition, some of these groups posted the survey link on their social media. Participation in both data acquisition waves was independent from each other.

### Measures

The online questionnaire contained sociodemographic questions (i.e. gender identity and sexual orientation, age, relationship status, place of living, and work status), questions on loneliness, depressive symptoms, social contacts, and health care.

*Gender identity and sexual orientation* were measured both as multiple answer categories; In the first data acquisition wave, it was one combined question (“Which of the following categories fit you best?”) with the options “asexual,” “bisexual,” “cis,” “woman,” “heterosexual,” “homosexual,” “inter,” “lesbian,” “man,” “non‐binary,” “pansexual,” “gay [German slang],” and “trans.” In the second data acquisition wave, this was split up into two separate questions (“Which of the following categories regarding sexual orientation fit you best?” and “Which of the following categories regarding gender identity fit you best?”) and “aromantic” and “queer” as new additional categories based on answers on open end questions in the first data acquisition wave.

*Loneliness* was measured by the De Jong Gierveld Short Scale (De Jong Gierveld & Tilburg, 2006). The De Jong Gierveld Short Scale is a brief, reliable, and valid measure overall, emotional, and social loneliness, where two subscales (i.e. emotional and social loneliness) consist of three items each. There are three positively (“I experience a general sense of emptiness”) and three negatively (“There are plenty of people that I can lean on in case of trouble”) formulated items. In a Dutch survey, the combined 6‐item loneliness scale reached a reliability of Cronbach's alpha between .71 and .76 (De Jong Gierveld & Tilburg, 2006). The reliability of the subscales in a German sample measured by Cronbach's alpha had been at least .83 for emotional loneliness and .91 for social loneliness (De Jong Gierveld & Van Tilburg, 2010). In our sample, Cronbach's alpha was calculated as .77. Answers were coded on a 4‐point Likert scale from 1 to 4 with higher values indicating an increased loneliness. Loneliness was calculated as mean value of all non‐missing items. A mean loneliness value larger than 2.5 was the cut‐off to be categorized as lonely as it has been previously used by Huxhold et al. (2019).

*Depressive symptoms* were measured by a validated 8‐item questionnaire on depressive symptoms in a non‐clinical context (Mohr & Müller, 2014): The scale is based on the depression model by Beck (King, 2002). It is a non‐clinical scale intended to measure impairments of well‐being with a preventive purpose. The scale consists of eight items with answers on a 7‐point Likert scale. The depressive symptoms scale ranges from 1 to 7 with 7 indicating poor mental health in terms of depressive symptoms. Reliability has been measured a Cronbach's alpha of .8 (Mohr & Müller, 2014). In our sample, Cronbach's alpha was calculated as .9.

*Social contacts*: Additionally, we asked for the number of face‐to‐face contacts last week before taking the survey as well as contacts via video or telephone call.

*Age*: In the questionnaire, we asked for age in 10‐year intervals, starting with the age group 18–25 years up until 76 years or older.

Regarding *health services utilization*, we asked if participants had a general practitioner (GP) and if they were currently in psychotherapy.

*Partner and parenthood status*: Respondents were asked if they currently have a partner. In the second wave, we additionally asked if they had underage children.

*Living environment*: We asked respondents for their current living environment with the categories “Rural,” “Small City,” “Medium‐Sized City,” and “Urban.”

### Data analysis

First, we performed descriptive analyses for all participants across cohorts. Then, the sample was split into two exclusive categories: LGBT persons (i.e. at least one of the categories: gay, lesbian, homosexual, bisexual, pansexual, asexual, aromantic, queer, trans, inter, non‐binary) or cis‐heterosexual persons (i.e. at least one of the categories man, woman, cis, heterosexual, and none of the LGBT categories). In a consecutive step, we conducted linear regression modeling with depressive symptoms as dependent variables. Different models have been calculated including LGBT status (Model 1), loneliness (Model 2), age (coded numerically as ordinal variable), number of face‐to‐face and video/telephone contacts and data acquisition wave (Model 4). As an alternative to Model 2, in Model 3 social and emotional loneliness have been entered into the model seperately. We conducted a mediation analysis with loneliness as mediating factor in the relation of being LGBT and depressive symptoms via loneliness using the “mediation” package in R.

Finally, as a sensitivity analysis, we conducted a propensity score matching between the first and second wave of data acquisition based on the variables age, living environment and LGBT status by using the “MatchIt” package in R (Model 5). As matching method, we used “nearest neighbor matching” with a 1:1 ratio.

As a second sensitivity analysis, we conducted the regression for each data acquisition wave separately. All analyses were conducted using R v4.0.2. Confidence intervals were calculated with 95%.

## RESULTS

### Participants

In total, 6748 participants took part in the survey: 2641 in the first data acquisition wave and 4143 in the second one. Of the participants of the second data acquisition wave, 108 stated they took part also in the first wave, 743 stated they were unsure, and 3139 stated not have taken part in the first wave.

Participants were mostly younger than 65 years with a peak in the age group 26 till 35 (cf. Figure S1a). Participants lived mainly in urban areas and to smaller shares in rural areas, small cities, or medium‐sized cities (cf. Figure S1b). Out of all participants, 5442 (80.6%) of the participants identified as LGBT, 1035 (15.3%) identified as cis‐heterosexual, and for 352 (5.2%), participants' information on gender identity and sexual orientation were missing. Regarding LGBT categories, most categories had more than 400 participants, and only inter and aromantic had lower counts (cf. Table 1). Concerning partner and children status, 3529 (59.4%) of participants had a partner: Only 57% of LGBT persons had a partner, but 73% of cis‐heterosexual persons had a partner; 424 (12.8%) of participants in the second data acquisition wave had children.

**TABLE 1:** Sexual orientation and gender identity in both waves of data acquisition

Category	Both waves of data acquisition	First wave of data acquisition	Second wave of data acquisition	Comparing first and second wave of data acquisition with *χ* ^2^ test
Man	2521	560	1961	*χ* ^2^(1) = 481.7, *p* < .001
Woman	2388	892	1496	*χ* ^2^(1) = 4.1, *p* = .042
Homosexual	2321	706	1615	*χ* ^2^(1) = 110.1, *p* = .001
Cis	1661	429	1232	*χ* ^2^(1) = 161, *p* < .001
Gay [German slang]	1521	479	1042	*χ* ^2^(1) = 46.3, *p* < .001
Lesbian	1143	442	701	*χ* ^2^(1) = 0.0, *p* = .831
Heterosexual	1047	517	530	*χ* ^2^(1) = 55.9, *p* < .001
Bisexual	969	368	601	*χ* ^2^(1) = 0.4, *p* = .505
Queer (orientation^a^)	949	‐^b^	949	‐
Queer (identity^a^)	841	‐^b^	841	‐
Non‐binary	737	306	431	*χ* ^2^(1) = 2.1, *p* = .147
Pansexual	737	310	427	*χ* ^2^(1) = 3.1, *p* = .076
Trans	601	205	396	*χ* ^2^(1) = 6.4, *p* = .011
Asexual	445	145	300	*χ* ^2^(1) = 8.0, *p* = .005
Aromantic	118	‐^b^	118	‐
Inter	39	16	23	*χ* ^2^(1) = 0.0, *p* = .923

^a^ Queer was an option in both categories.

^b^ The options queer and aromantic have been added for the second data acquisition wave.

### Loneliness

The mean of loneliness was 2.32 with a median of 2.33. The mean of social loneliness was 1.97, and the median was 2. The mean of emotional loneliness was 2.67, and the median was 2.67. The correlation between both subscales was *r* = .41.

One‐third of the participants had a loneliness score higher than 2.5 points and thus qualified as lonely (*n* = 1938). There was no clear association of loneliness with age (cf. Table 2); however, in all age groups, LGBT participants were in mean more lonely than cis‐heterosexual participants but not significantly from 55 years on. Loneliness was higher in the mean of 2.37 (95% CI [2.35, 2.39]) in the second data acquisition wave compared with the first data acquisition wave with a mean of 2.24 (95% CI [2.21, 2.26], *t*(4732.2) = −8.3, *p* < .001). Loneliness was significantly lower in persons with a partner with a mean value of 2.16 (95% CI [2.14, 2.18]) compared with persons without a partner with a value of 2.55 (95% CI [2.53, 2.57]; *t*(4984.5) = −25.1, *p* < .001).

**TABLE 2:** Mean loneliness with 95% CI by age group and LGBT status on a scale ranging from 1 to 4 with higher values as higher loneliness

	All participants	LGBT participants	Cis‐heterosexual participants	Students *t*‐test comparing LGBT and cis‐heterosexual participants
Age group				
18–25 years	2.40 [2.36, 2.43]	2.46 [2.42, 2.49]	2.07 [2.00, 2.15]	*t*(313.5) = 9.2, *p* < .001
26–35 years	2.31 [2.28, 2.33]	2.36 [2.33, 2.38]	2.04 [1.97, 2.11]	*t*(430.5) = 8.4, *p* < .001
36–45 years	2.29 [2.25, 2.32]	2.31 [2.27, 2.35]	2.16 [2.08, 2.24]	*t*(318.0) = 3.4, *p* < .001
46–55 years	2.31 [2.27, 2.35]	2.34 [2.30, 2.39]	2.05 [1.95, 2.15]	*t*(146.2) = 5.2, *p* < .001
56–65 years	2.33 [2.27, 2.39]	2.36 [2.29, 2.43]	2.16 [2.02, 2.30]	*t*(90.8) = 2.5, *p* = .015
66 years or older	2.08 [1.97, 2.19]	2.11 [1.98, 2.24]	1.88 [1.69, 2.08]	*t*(40.3) = 2.0, *p* = .051

Abbreviation: LGBT, lesbian, gay, bisexual, trans, inter, asexual, and queer.

Loneliness was highly dependent on sexual orientation and gender identity (cf. Table 3 for subgroups) with lower proportions of loneliness for cis‐heterosexual persons compared with LGBT persons. In all groups, the proportion of lonely persons was higher in persons with partner compared with those without partner; this difference was significant in all groups but aromantic, asexual and inter persons.

**TABLE 3:** Relative frequency of lonely participants by sexual orientation and gender identity and partner status with 95% CI

Category	All participants	Participants with a partner	Participants without a partner	Comparing participants with and without a partner with *χ* ^2^‐test
Cis‐heterosexual^a^	19.5%[17.0%, 22.2%]	16.1% [13.5%,19.2%]	27.5% [22.0%, 33.7%]	*χ* ^2^(1, *n* = 903) = 14.0, *p* < .001
Heterosexual	21.3% [18.8%, 24.1%]	17.9% [15.1%, 21.0%]	29.6% [24.2%, 35.6%]	*χ* ^2^ (1, *n* = 926) = 14.8, *p* < .001
Cis	30.1% [27.8%, 32.5%]	19.1% [16.6%, 22.0%]	45.4% [41.4%, 49.4%]	*χ* ^2^ (1, *n* = 1486) = 117.1, *p* < .001
Woman	30.3% [28.3%, 32.2%]	21.9% [19.8%, 24.2%]	44.6% [41.0%, 48.2%]	*χ* ^2^ (1, *n* = 2123) = 118.4, *p* < .001
Homosexual	32.5% [30.5%, 34.6%]	20.1% [17.9%, 22.5%]	49.2% [45.8%, 52.6%]	*χ* ^2^ (1, *n* = 2046) = 191.5, *p* < .001
Gay [German slang]	32.8% [30.3%, 35.4%]	21.6% [18.7%, 24.8%]	47.1% [43.0%, 51.2%]	*χ* ^2^ (1, *n* = 1340) = 96.4, *p* < .001
Man	33.5% [31.6%, 35.5%]	22.0% [19.8%, 24.4%]	48.9% [45.7%, 52.1%]	*χ* ^2^ (1, *n* = 2229) = 176.1, *p* < .001
Lesbian	33.5% [30.6%, 36.5%]	21.6% [18.5%, 25.1%]	53.5% [48.3%, 58.6%]	*χ* ^2^ (1, *n* = 1012) = 106.1, *p* < .001
LGBT^b^	35.5% [34.1%, 36.9%]	24.2% [22.6%, 25.9%]	50.2% [48.0%, 52.3%]	*χ* ^2^ (1, *n* = 4780) = 344.1, *p* < .001
Bisexual	37.6% [34.4%, 40.9%]	29.5% [25.5%, 33.7%]	48.1% [42.9%, 53.3%]	*χ* ^2^ (1, *n* = 869) = 30.8, *p* < .001
Pansexual	42.2% [38.5%, 46.1%]	34.1% [29.5%, 39.1%]	53.6% [47.4%, 59.7%]	*χ* ^2^ (1, *n* = 655) = 23.8, *p* < .001
Queer (identity^c^)	42.3% [38.7%, 45.9%]	29.8% [25.5%, 34.5%]	57.4% [51.9%, 62.8%]	*χ* ^2^ (1, *n* = 747) = 56.5, *p* < .001
Queer (orientation^c^)	42.3% [39.0%, 45.7%]	30.9% [26.9%, 35.4%]	56.2% [51.1%, 61.2%]	*χ* ^2^ (1, *n* = 861) = 54.7, *p* < .001
Aromantic	42.7% [33.5%, 52.5%]	39.1% [20.5%, 61.2%]	43.5% [32.9%, 54.7%]	*χ* ^2^ (1, *n* = 108) = 0.0, *p* = .888
Non‐binary	48.2% [44.4%, 52.1%]	40.5% [35.3%, 45.9%]	57.1% [51.5%, 62.6%]	*χ* ^2^ (1, *n* = 661) = 17.7, *p* < .001
Trans	48.8% [44.6%, 53.1%]	38.8% [33.2%, 44.6%]	61.5% [55.0%, 67.6%]	*χ* ^2^ (1, *n* = 538) = 26.6, *p* < .001
Asexual	51.7% [46.8%, 56.6%]	44.7% [35.8%, 53.9%]	55.2% [49.3%, 61.1%]	*χ* ^2^ (1, *n* = 409) = 3.4, *p* = .065
Inter	54.1% [37.1%, 70.2%]	47.8% [27.4%, 68.9%]	61.5% [32.3%, 84.9%]	*χ* ^2^ (1, *n* = 36) = 0.2, *p* = .657

Abbreviation: LGBT, lesbian, gay, bisexual, trans, inter, asexual, and queer.

^a^ Constructed category.

^b^ Constructed category.

^c^ Queer was an option in both categories.

### Depressive symptoms

The distribution of depressive symptoms was slightly right skewed (skewness 0.17) with a mean of 3.57 and a median of 3.5. Mean depressive symptoms were lower in older age groups and were higher for LGBT participants than cis‐heterosexual participants in each age group but not significantly from 55 years on (cf. Table S1). Levels of depressive symptoms were elevated in the second data acquisition wave than in the first data acquisition wave with a mean of 3.40, 95% CI [3.35, 3.45] in the first and a mean of 3.68, 95% CI [3.64, 3.73] in the second data acquisition wave (*t*(5051.5) = −8.5, *p* < .001).

The level of depressive symptoms varied according to sexual orientation and gender identity (cf. Table S2 for subgroups) with lower levels of depressive symptoms for cis‐heterosexual persons compared with LGBT persons: Trans, pansexual, asexual, non‐binary, and aromantic persons showed the highest mean levels of depressive symptoms.

### Social contacts

The number of face‐to‐face contacts in the week prior to the survey ranges from 0 to 145 contacts. With a mean of 8.07 and a median of 5 contacts, the distribution is highly right skewed (skewness 4.00). The mean number of face‐to‐face contacts decreased from 8.45 (95% CI [8.05, 8.85]) contacts in the first data acquisition wave to 7.83 (95% CI [7.50, 8.17]) in the second data acquisition wave (*t*(5186.2) = 2.3, *p* = .02).

The number of video or telephone contacts in the week prior to the survey ranges from 0 to 200 contacts. With a mean of 4.62 and a median of 3 contacts, the distribution is highly right skewed (skewness 9.52). The mean number of video or telephone contacts was higher in the second data acquisition wave with 4.77 (95% CI [4.51, 5.03]) compared with 4.39 (95% CI [4.13, 4.65]) contacts in the first data acquisition wave (*t*(5687.4) = −2.0, *p* = .046).

The Spearman correlation coefficient between face‐to‐face contacts and video or telephone contacts is *r* = .15.

### Depressive symptoms, loneliness, and being LGBT

In the basic model (Model 1, cf. Table 4), being LGBT was related with increased depressive symptoms by 0.53 (*p* < .001, 95% CI [0.44, 0.61]) points. However, including loneliness into the model (Model 2), the effect of being LGBT weakens to 0.20 (*p* < .001, 95% CI [0.14, 0.28]) points, whereas one point on the loneliness scale increases depressive symptoms by 1.18 (*p* < .001, 95% CI [1.13, 1.23]) points. As a sensitivity check, splitting the loneliness scale into the two subscales (Model 3) does not change the effect of being LGBT (0.21, *p* < .001, 95% CI [0.14, 0.29]); emotional loneliness has with 0.80 (*p* < .001, 95% CI [0.76, 0.84]) points a larger impact than social loneliness with 0.38 (*p* < .001, 95% CI [0.34, 0.42]) points.

**TABLE 4:** Standardised linear regression coefficients on depressive symptoms scale with 95% CI

Variable	Model 1	Model 2	Model 3	Model 4	Model 5 (matched)
LGBT	0.15***, [0.13, 0.18]	0.06***, [0.04, 0.08]	0.06***, [0.04, 0.08]	0.06***, [0.04, 0.08]	0.08***, [0.05, 0.10]
Loneliness	‐	0.58***, [0.56, 0.60]	‐	0.54***, [0.52, 0.56]	0.53***, [0.51, 0.55]
Social loneliness	‐	‐	0.22***, [0.30, 0.25]	‐	‐
Emotional loneliness	‐	‐	0.46***, [0.44, 0.48]	‐	‐
Wave	‐	‐	‐	0.06***, [0.04, 0.09]	0.09***, [0.07, 0.11]
Face‐to‐face contacts	‐	‐	‐	−0.05***, [−0.08, −0.03]	−0.06***, [−0.08, −0.04]
Video/telephone contacts	‐	‐	‐	−0.04***, [−0.06, −0.01]	−0.03*, [−0.06, −0.01]
Age group	‐	‐	‐	−0.23***, [−0.21, −0.25]	−0.23***, [−0.20, −0.25]
*R* ^2^	.02	.35	.36	.41	.41

Abbreviation: LGBT, lesbian, gay, bisexual, trans, inter, asexual, and queer.

* *p* < .05.

** *p* < .01.

*** *p* < .001.

**FIGURE 1:**
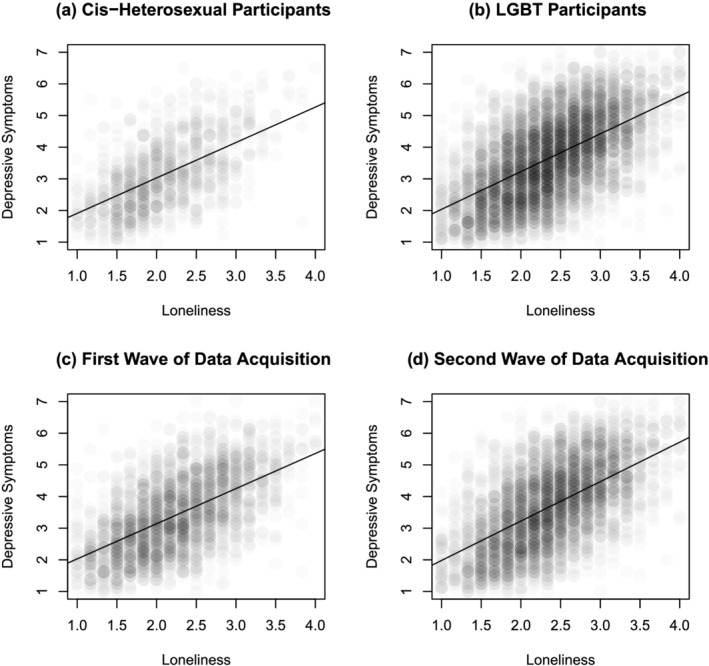
Association between loneliness and depressive symptoms with result of unadjusted linear regression separately for cis‐heterosexual participants (a) and LGBT participants (b) as well as the first data acquisition wave (c) and the second data acquisition wave (d)

In the fully adjusted model (Model 4), controlling for age, face‐to‐face contacts, video/telephone contacts, and the data acquisition wave, the effect of being LGBT is unchanged with 0.20 (*p* < .001, 95% CI [0.13, 0.27]) points, and the effect of loneliness is unchanged as well with 1.11 (*p* < .001, 95% CI [1.07, 1.15]) points. Depressive symptoms increase with data acquisition wave by 0.17 (*p* < .001, 95% CI [0.11, 0.22]) points and decreases with each age group by 0.23 (*p* < .001, 95% CI [0.21, 0.25]) points. Figure 1 shows the consistent relationship between loneliness and depressive symptoms for LGBT and cis‐heterosexual persons as well as in both data acquisition waves. Mediation analysis with nonparametric bootstrap estimates showed a total effect of LGBT on depressive symptoms of −0.54 (*p* < .001, 95% CI [−0.62, −0.45]). Introducing loneliness as a mediator to the model and thereby decomposing the total effect, the average mediation effect from LGBT via loneliness on depressive symptoms was −0.33 (*p* < .001, 95% CI [−0.38, −0.28]), whereas the remaining average direct effect of LGBT on depressive symptoms was −0.21 (*p* < .001, 95%, CI [−0.28, −0.14]). Thus, 61% of the effect of being LGBT on depressive symptoms was mediated by loneliness.

The *R* ^2^ increased from Model 1 to Model 2 dramatically from .02 to .35 with a further increase with Model 4 to .41. The Akaike Information Criterion (AIC) improved accordingly from Model 1 with 16,180 over Model 2 with 13,712 and Model 3 with 13,559 to Model 4 with 13,016.

In the sensitivity analysis with propensity score matched data (Model 5), the effect of the data acquisition wave increased slightly to 0.22 (*p* < .001, 95% CI [0.17, 0.28]) leaving the other covariates in about the same order.

## DISCUSSION

Investigating the relation between loneliness and depressive symptoms in those being LGBT compared with cis‐heterosexual persons during the first year of the pandemic, our results have shown elevated levels of loneliness from the beginning to later during the COVID‐19 pandemic while physical distancing measures in Germany were in place (Hypothesis 1). Loneliness was especially high in LGBT persons, particularly in asexual, trans, and non‐binary persons (Hypothesis 2) and LGBT persons without a partner. We found a linear association between loneliness and depressive symptoms, even when adjusting for social contacts, which was comparable for LGBT and cis‐heterosexual persons (Hypothesis 3). Loneliness mediated 61% of the association between being LGBT and depressive symptoms (Hypothesis 4). To the existing literature, these results add especially the mediating role of loneliness and the proof of vulnerability of LGBT regarding physical distancing measures.

In our study, loneliness and depressive symptoms are higher in all subgroups than in representative surveys of the German population before the COVID‐19 pandemic (De Jong Gierveld & Van Tilburg, 2010; Mohr & Müller, 2014). The results are in line with findings of an increased loneliness both in LGBT persons before the COVID‐19 pandemic (Fredriksen‐Goldsen et al., 2013; Hsieh & Liu, 2021) and an increased loneliness of the general population during the COVID‐19 pandemic. Our study adds that LGBT persons were especially lonely during the pandemic, with extraordinary high loneliness scores for asexual, trans, and non‐binary persons. This is in line with previous literature (Borgogna et al., 2019; McInroy et al., 2020). In addition, the present study illuminates an important role of loneliness in the association between depressive symptoms and sexual orientation/gender identity. Future research is needed to gain a better understanding of the mediating role of loneliness in mental health of LGBT individuals.

The minority stress model by Meyer (1995) gives an explanatory framework for our results. Internalized homonegativity, stigmatization, and perceived discrimination, which constitute minority stress interfere with everyday social interaction for individuals from the LGBT community (Meyer, 1995). For instance, Kuyper (2010) could show that minority stress is associated with increased levels of loneliness of elder gay men in the Netherlands: while older adults with a larger LGB network felt less socially lonely, factors like previous experience of or expectation of negative reactions led to high levels of loneliness (Kuyper & Fokkema, 2010). McInroy et al. (2020) showed asexual youth to have lower mental health and a significantly higher internalized LGBTQ‐phobia than non‐asexual individuals, and social stigma surrounding gender non‐conformity as well as loneliness have been described to negatively impact mental health of transgender men and women (Bockting et al., 2016; Fernandez‐Rouco et al., 2019; McInroy et al., 2020). Thus, it seems plausible with the existing literature that the minority stress of LGBT persons leads to an increased risk for loneliness, which is, in turn, associated with depressive symptoms. This is especially in line with our findings that LGBT persons without a partner have the strongest risk for loneliness. Future research should address the association of minority stress and loneliness explicitely.

Fish et al. (2020) and Salerno et al. (2020) described how younger persons are affected by loneliness during the pandemic: Social and physical distancing measures forced LGBT youth into challenging and possibly unsupportive living conditions, leading to an increased risk of family rejection, harassment, and mental health problems, while at the same time losing access to community and institutional support systems (Fish et al., 2020; Salerno et al., 2020). These findings are also in line with Gonzales et al. (2020), reporting that 45.7% of LGBT college students have families not supportive or unaware of their identity and 60% experiencing mental health problems during the pandemic (Gonzales et al., 2020). To sum up, present findings outline a particularly important role of loneliness for the mental health of LGBT individuals.

### Strengths and limitations

Our study has strengths and limitations. A particular strength is the large sample size of usually underrepresented LGBT persons. For Germany, the sample of the German Socio‐Economic Panel (GSOEP) (SOEP, 2020) constitutes a notable exception, but trans and asexual persons are not represented either, making our study a valuable contribution to the literature on mental health in LGBT persons. Further, our survey took place at the peaks of COVID‐19 waves in Germany, while most studies were just retrospectively in nature due to the abruptness of the pandemic. Lastly, the measurement instrument assessing sexual orientation and identity was without restricting the participants to just one answer and was fully self‐categorizing, which allowed a high acceptability in the community.

Limitations need to be mentioned as well. The sampling strategy was oversampling LGBT persons, but sample was not at all representative for the general population. Further, due to the anonymous study design, we were not able to follow up participants but used a cohort design only. Although we used propensity score matching to harmonize samples across waves for a sensitivity analysis, intraindividual hypotheses could not be addressed and need to be investigated in future studies on disasters and minority stress. Education and socioeconomic status might play a role regarding loneliness and depressive symptoms, however were not included in the questionnaire. Finally, mediation analysis was not used as a test of causal effect rather than as a heuristic tool, causal relations between LGBT persons, loneliness and depressive symptoms should be tested in future interventional research.

The study has been conducted in Germany, a country where discrimination against LGBT persons is forbidden, however still happening on a daily basis. Thus, the results of our study are mainly transferable to western countries with a similar situation. In many countries of the world, LGBT persons face public prosecution. Hence, one might expect a much higher minority stress level of LGBT persons in such countries.

## CONCLUSIONS

Our results point at future interventions to enhance mental health during physical distancing measures and for LGBT persons. The substantial mediation effect of loneliness on depression indicates that loneliness might be a good target for future intervention studies. Loneliness can be influenced by interventions as studies in primary care settings have been shown to be effective (Gardiner et al., 2018; Rodríguez‐Romero et al., 2021).

The results highlight the importance of loneliness during physical distancing measures. Digital interventions that do not rely on physical contact may offer support during physical distancing measures (Craig et al., 2020; Pachankis et al., 2020) and might be well accepted by LGBT persons (Peterson et al., 2020). Such digital interventions could be established widely and made accessible especially for minorities that are more at risk for negative mental health impacts.

On a general level, our results indicate that physical distancing measures during pandemic management should be re‐evaluated also by psychologists regarding possible negative side effects for minority groups in the society.

## CONFLICT OF INTEREST

Pichit Buspavanich received a research grant from Gilead. The other authors declare to have no competing financial interest to declare.

## ETHICS STATEMENT

The study adhered to the Declaration of Helsinki. All participants gave their informed consent before taking part in the online survey. Data acquisition was anonymous without identifying variables. Because of the anonymity of the study, the consultation of an ethical review board was not necessary. Participants did not receive any kind of incentive or remuneration.

## Supporting information


**Table S1.** Mean Depressive Symptoms With 95% CI by Age Group and LGBT‐Status on a Scale Ranging From 1 to 7 With Higher Values as Higher Depressive Symptoms

**Table S2.** Mean Depressive Symptoms With 95% CI by Sexual Orientation and Gender Identity and Waves of Data Acquisition

**Figure S1:** Age distribution and living environment of the sample (*N* = 6,748) split by LGBT‐status

## Data Availability

Data are available on request if the request is in line with the informed consent of the study.
