# Supplementary material for: Loneliness and depressive symptoms differ by sexual orientation and gender identity during physical distancing measures in response to COVID‐19 pandemic in Germany
Source: Appl Psychol Health Well Being. 2022 Jun 6:10.1111/aphw.12376. Online ahead of print. doi: 10.1111/aphw.12376 (PMC9348355; doi:10.1111/aphw.12376)
Supplement: Supplementary file 1 — Table S1. Mean Depressive Symptoms With 95% CI by Age Group and LGBT‐Status on a Scale Ranging From 1 to 7 With Higher Values as Higher Depressive Symptoms [file APHW-9999-0-s001.docx]

Table S1

*Mean Depressive Symptoms With 95% CI by Age Group and LGBT-Status on a Scale Ranging From 1 to 7 With Higher Values as Higher Depressive Symptoms*

|  | All participants | LGBT participants | Cis-heterosexual participants | Student’s t-test comparing LGBT and cis-heterosexual participants |
| --- | --- | --- | --- | --- |
| Age group |  |  |  |  |
| 18-25 years | 4.07 [4.00, 4.13] | 4.20 [4.14, 4.27] | 3.38 [3.23, 3.53] | *t*(313.1) = 9.8, *p* < .001 |
| 26-35 years | 3.68 [3.62, 3.73] | 3.77 [3.72, 3.83] | 3.18 [3.05, 3.30] | *t*(460.7) = 8.7, *p* < .001 |
| 36-45 years | 3.38 [3.31, 3.45] | 3.42 [3.35, 3.49] | 3.11 [2.95, 3.28] | *t*(299.3) = 3.3, *p* < .001 |
| 46-55 years | 3.17 [3.09, 3.25] | 3.21 [3.12, 3.31] | 2.78 [2.59, 2.96] | *t*(160.8) = 4.2, *p* < .001 |
| 56-65 years | 3.18 [3.06, 3.31] | 3.23 [3.10, 3.37] | 2.85 [2.58, 3.11] | *t*(90.7) = 2.6,  *p* = .012 |
| 66 years or older | 2.86 [2.66, 3.07] | 2.93 [2.70, 3.16] | 2.42 [1.97, 2.88] | *t*(30.2) = 2.0,  *p* = .051 |
