# Supplementary material for: Loneliness and depressive symptoms differ by sexual orientation and gender identity during physical distancing measures in response to COVID‐19 pandemic in Germany
Source: Appl Psychol Health Well Being. 2022 Jun 6:10.1111/aphw.12376. Online ahead of print. doi: 10.1111/aphw.12376 (PMC9348355; doi:10.1111/aphw.12376)
Supplement: Supplementary file 2 — Table S2. Mean Depressive Symptoms With 95% CI by Sexual Orientation and Gender Identity and Waves of Data Acquisition [file APHW-9999-0-s002.docx]

Table S2

*Mean Depressive Symptoms With 95% CI by Sexual Orientation and Gender Identity and Waves of Data Acquisition*

| Category | First wave of data acquisition | Second wave of data acquisition | Student’s t-test comparing first and second wave of data acquisition |
| --- | --- | --- | --- |
| Cis-heterosexual^a^ | 3.00 [2.91, 3.08] | 3.31 [3.18, 3.43] | *t*(749.6) = -4.0,  *p* < .001 |
| Heterosexual | 3.02 [2.92, 3.12] | 3.43 [3.32, 3.54] | *t*(918.9) = -5.5,  *p* < .001 |
| Man | 3.37 [3.26, 3.47] | 3.49 [3.43, 3.55] | *t*(856.2) = -1.9,  *p* = .053 |
| Gay [German slang] | 3.24 [3.13, 3.35] | 3.54 [3.46, 3.62] | *t*(870.8) = -4.3,  *p* < .001 |
| Homosexual | 3.28 [3.20, 3.38] | 3.54 [3.47, 3.60] | *t*(1,283.3) = -4.4, *p* < .001 |
| Lesbian | 3.43 [3.32, 3.55] | 3.66 [3.56, 3.76] | *t*(907.1) = -2.9,  *p* = .004 |
| LGBT^a^ | 3.51 [3.45, 3.57] | 3.73 [3.68, 3.77] | *t*(3,580.1) = -5.9, *p* < .001 |
| Woman | 3.34 [3.27, 3.41] | 3.77 [3.70, 3.83] | *t*(1,877.2) = -8.3, *p* < .001 |
| Cis | 3.43 [3.32, 3.53] | 3.80 [3.73, 3.87] | *t*(740.8) = -5.8,  *p* < .001 |
| Queer (Identity^b^) | - | 3.92 [3.83, 4.01] | - |
| Queer (Orientation^b^) | - | 4.04 [3.96, 4.12] | - |
| Inter | 2.91 [2.24, 3.58] | 4.11 [3.49, 4.73] | *t*(33.6) = -2.8,  *p* = .009 |
| Bisexual | 3.68 [3.56, 3.80] | 4.13 [4.03, 4.24] | *t*(731.3) = -5.7,  *p* < .001 |
| Asexual | 3.99 [3.80, 4.19] | 4.22 [4.07, 4.36] | *t*(274.4) = -1.8,  *p* = .072 |
| Pansexual | 3.96 [3.82, 4.09] | 4.26 [4.14, 4.37] | *t*(604.5) = -3.3,  *p* = .001 |
| Aromantic | - | 4.29 [4.05, 4.52] | - |
| Trans | 3.74 [3.58, 3.90] | 4.30 [4.17, 4-43] | *t*(420.9) = -5.4,  *p* < .001 |
| Non-Binary | 4.04 [3.91, 4.18] | 4.36 [4.24, 4.47] | *t*(584.1) = -3.5,  *p* < .001 |

^a^Constructed category

^b^Queer was an option in both categories
