# Supplementary figures and images for: Loneliness and depressive symptoms differ by sexual orientation and gender identity during physical distancing measures in response to COVID‐19 pandemic in Germany
Source: Appl Psychol Health Well Being. 2022 Jun 6:10.1111/aphw.12376. Online ahead of print. doi: 10.1111/aphw.12376 (PMC9348355; doi:10.1111/aphw.12376)

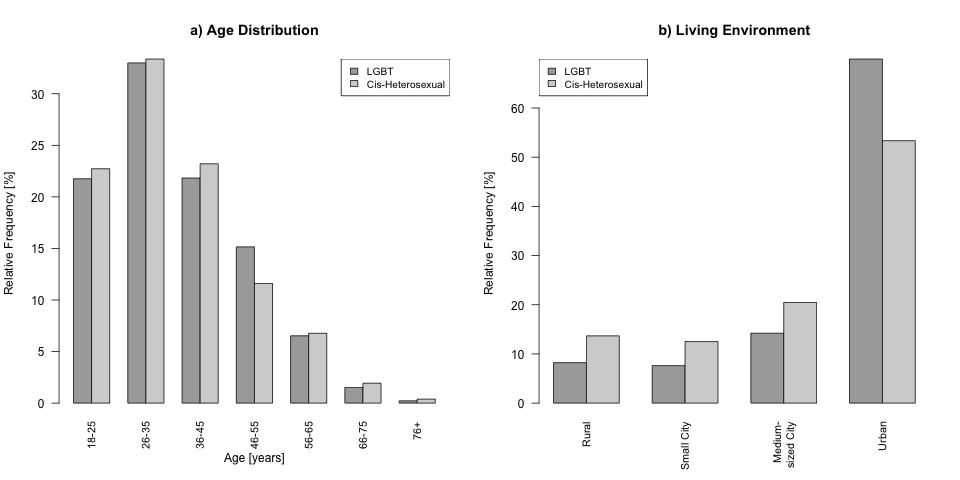

Supplement: Supplementary file 3 — Figure S1: Age distribution and living environment of the sample (N = 6,748) split by LGBT‐status [file APHW-9999-0-s003.png]
